# The Clinical Use of the Suppression Head Impulse Paradigm in Patients with Vestibulopathy: A Systematic Review

**DOI:** 10.3390/healthcare10071182

**Published:** 2022-06-24

**Authors:** Leonardo Manzari, Sara De Angelis, Alessandro Antonio Princi, Giovanni Galeoto, Marco Tramontano

**Affiliations:** 1 MSA ENT Academy Center, 03043 Cassino, Italy; 2 Fondazione Santa Lucia IRCCS, 00179 Rome, Italy; s.deangelis@hsantalucia.it (S.D.A.); a.princi@hsantalucia.it (A.A.P.); m.tramontano@hsantalucia.it (M.T.); 3 Department of Human Neurosciences, Sapienza University of Rome, 00185 Rome, Italy; giovanni.galeoto@uniroma1.it; 4 Department of Movement, Human and Health Sciences, University of Rome “Foro Italico”, 00135 Rome, Italy

**Keywords:** vestibulopathy, vestibular system, SHIMP, HIMP, vHIT

## Abstract

Background: This review aims to explore the potential clinical application of the suppression head impulse paradigm (SHIMP) in patients with unilateral and bilateral vestibulopathy. Methods: An electronic search was conducted by two independent reviewers in the following databases: Embase, MEDLINE (PubMed), and Scopus. The screening of titles, abstracts, and full texts and data extraction were undertaken independently by pairs of reviewers. The included studies were quality appraised using a modified version of the Newcastle–Ottawa Scale. Results: The results were reported following the PRISMA (Preferred Reporting Items for Systematic Reviews and Meta-Analyses). Our search yielded 935 unique records, of which 16 remained after screening titles and abstracts. A total of 11 studies were included, covering a total of 418 participants (230 patients and 188 healthy participants). Conclusion: SHIMP could be a useful tool to diagnose a VOR alteration in patients with vestibulopathy in both the acute and chronic phases of vestibulopathy.

## 1. Introduction

The oculomotor response to impulsive rotation of the head represents the output for a short latency brainstem reflex that originates from the activation of the semicircular canals. This phylogenetically old reflex, the vestibular ocular reflex (VOR), consists of a disynaptic elementary pathway that allows the task of stabilizing the images on the retina during the movements of the head in the activities of daily living [1]. However, despite its simplicity, this reflex arc is anything but stereotyped. Rather, its response characteristics have been shown to be extraordinarily adaptable to behavioral needs [2].

The instrumental assessment of the vestibular system has made significant progress in recent years.

Two protocol tests are available in the clinical practice to evaluate the VOR function through the use of the video head impulse test (vHIT): the head impulse paradigm (HIMP) and the suppression head impulse paradigm (SHIMP) [3]. These tests can be used alone (in the case of HIMP) or in combination to test semicircular canal function and to determine the residual VOR gain and the visuo-vestibular interaction [4].

The basic physiology underlying both the HIMP and the SHIMP is the fluid displacement in the semicircular canals, which deflects the hair bundles of receptor hair cells, generating coordinated eye movements to stabilize the gaze during an unpredictable head turn [5].

The head turn stimulus and the eye movement recording are identical. All that is changed are the instructions—from “look at that fixed target on the wall” to “look at the moving target”.

For the SHIMP test execution, during the passive head rotation the patient is instructed to look at a head-fixed target rather than the earth-fixed target utilized in the HIMP. Patients can understand the test paradigm much easier if they glance at the dot. The person does not realize he is making a saccade.

Compared to the HIMP, in patients with vestibulopathy the SHIMP shows a reversed saccadic pattern: HIMPs elicit compensatory saccades opposite to the direction of head rotation. In contrast, the SHIMP elicits anti-compensatory saccades in the direction of head rotation in healthy people. During the SHIMP protocol, patients with a VOR alteration in the very acute stage do not make corrective saccades, manifesting a vestibular hypofunction, whereas in the subacute or chronic stage of vestibular hypofunction, SHIMP anti-compensatory saccades indicate visuo-vestibular interaction and the recovery of residual vestibular function [4].

It is important to note that SHIMP saccades do not mean that there is a VOR suppression. Indeed, as Crane and Demer showed [6] in healthy people, VOR is fully operational during the latency time. For this reason, the VOR gains in HIMPs and SHIMPs are similar.

Both paradigms have the same basic physiology and can be characterized by two parameters: VOR gain and corrective or catch-up saccades. Before the SHIMP become available, the main parameters were VOR gain and the presence of covert and overt saccades, whereas from the development of the SHIMP, for the clinician, it is possible to better quantify the real value of VOR gain since it is rarely affected by the presence of covert saccades [4,7]. Furthermore, in individuals with vestibular neuritis (VN), substantial relationships between the HIMP and SHIMP quantitative characteristics were recently found [7]. Both HIMP gains (0.76) and SHIMP gains (0.66) showed 100% sensitivity and 100% specificity in identifying patients with BVL from normal controls. Similarly, with 100% sensitivity and 100% specificity, HIMP gains (0.76) and SHIMP gains (0.66) identified the affected side of UVL [3].

The SHIMP reveals two important advantages vs. the HIMP: first, it allows the measurement of the VOR gain slow phase, and second, the percentages of impulses containing SHIMP saccades in the affected side are reported [4]. In a healthy person whose head is abruptly rotated, the VOR will successfully maintain the eyes on an earth-stationary target (reflected by an absence of compensatory saccades on the HIMP and the presence of anti-compensatory saccades on the SHIMP); in contrast, in a patient with acute uncompensated unilateral vestibulopathy whose head is abruptly rotated towards the lesioned side, the eyes will be “dragged along” with the head such that the eyes remain on a head-fixed target (reflected by the presence of compensatory saccades on the HIMP and the absence of anti-compensatory saccades on the SHIMP).

The reappearance of saccades in the SHIMP is linked to the (even minimal) increase in VOR gain. The early vestibular input keeps the eyes on the original target location, but since the target has moved with the head, a visual error signal triggers a saccade to the updated target location. This phenomenon could be the manifestation of adaptive neural plasticity overcoming a deficit, providing information about the recovery through a visuo-vestibular interaction strategy in patients with VN.

To the best of our knowledge, no studies have systematically reported the clinical usefulness of the SHIMP during clinical practice in patients with vestibulopathy. For this reason, this review aims to explore the use of the SHIMP of the vHIT in vestibulopathy patients in both the acute and chronic phases compared to other vestibular instrumental assessments.

## 2. Materials and Methods

The preferred reporting elements for systematic reviews and meta-analyses (PRISMA) [8] were used to conduct the review. There was no protocol review registration.

### 2.1. Eligibility Criteria for Study Selection

No restrictions in the enrollment of participants due to socio-demographic conditions, gender, or age were applied. The inclusion criteria were: (1) participant—patients with unilateral and/or bilateral vestibulopathy; (2) intervention—the application of the SHIMP of the vHIT; (3) comparison—the HIMP of the vHIT and/or other vestibular tests; and (4) outcome—the usefulness of SHIMP to diagnose an alteration in unilateral and bilateral vestibulopathy patients in both the acute and chronic phases. Only studies written in English were included, and no year of publication restriction was adopted.

### 2.2. Information Sources

The electronic databases searched in December 2021 included Embase, MEDLINE (PubMed), and Scopus. The following search terms were used: (“head impulse test” OR “head impulse paradigm*” OR “suppression head impulse test” OR “suppression head impulse paradigm”). Search terms were modified for each database and appropriate subheadings were used for each searched database. We also performed a manual search of the bibliographies of eligible articles.

### 2.3. Study Selection and Data Collection Process

Duplicate records were found and deleted using EndNOTE software. After duplicates were removed, two investigators (AAP and SDA) independently extracted study data using a pre-specified data collection form. Discrepancies were discussed with a third reviewer (MT) to reach a consensus. The authors identified the studies that had to be further examined for inclusion in the review. Cohen’s kappa for the inter-reviewer agreement was 0.81, which indicated an almost perfect agreement.

### 2.4. Data Synthesis and Methodological Quality Assessment

The methodological quality of the evidence was assessed with a modified version of the Newcastle–Ottawa Scale (NOS) [9,10,11] (Appendix A) in which the maximum score that can be achieved is 7, i.e., 2 points on the selection subscale, 2 points on the treatment subscale, and 3 points on the outcome subscale.

## 3. Results

**Figure 1:**
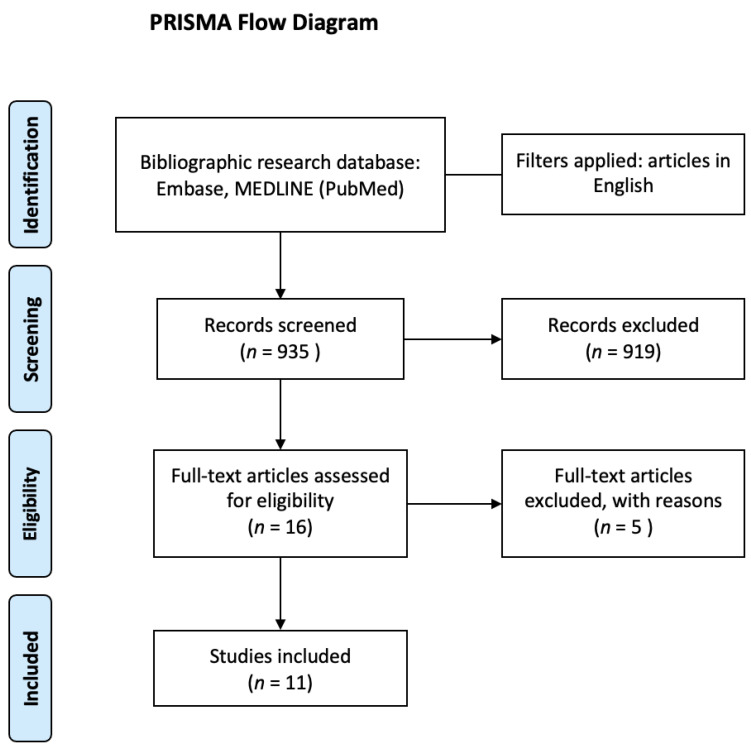
PRISMA flow diagram.

Our search yielded 935 unique records, of which 16 remained after screening titles and abstracts. We reviewed the full texts of these studies for eligibility and excluded five studies (three articles were in Chinese, and two articles were not available). Finally, 11 studies were included (Figure 1), covering a total of 418 participants (230 patients and 188 healthy participants).

For vHIT, different tools were used. Nine studies [4,7,12,13,14,15,16,17,18] used an Otometrics ICS impulse, one study [19] used an EyeSeeCamTM System, and one study [3] used a FireflyMV, Point Grey Research Inc. All instruments were validated with the gold standard scleral search coils. Three studies [4,7,12] evaluated patients within the first 3 days after the acute vestibular syndrome, and five studies evaluated patients in the sub-acute and chronic phases [13,15,17,18,19]. Two articles did not report the timing of the evaluation [16,20], and one study reported that the evaluation was performed in the acute phase without specifying the days since the onset [12]. The included studies enrolled patients with both unilateral (UV) and bilateral vestibulopathy (BV). The details are reported in Table 1.

The included studies all had methodological qualities ranging from poor to excellent, as indicated by the NOS scores presented in Table 1. Three studies reported scores of 7, four studies reported scores of 6, one study reported a score of 5, two studies reported scores of 4, and one study reported a score of 3.

In total, 9 out of 11 studies included patients with UV. In particular, five studies enrolled 86 patients with UV after vestibular neuritis (VN) [4,7,14,15,19], two studies enrolled 20 patients with UV after Menière’s disease [18,19], two studies included 43 patients with UV after treatment of vestibular schwannoma [3,18], and two studies enrolled UV patients without specifying the diagnosis [12,17]. In addition, 6 out of 11 studies enrolled patients with BV. In particular, one study included a Lyme Disease patient with BV [13], five studies enrolled 16 patients with idiopathic BV [3,15,16,18,19], one study included 1 patient with genetic BV [16], and two studies enrolled 5 patients with BV after systemic gentamicin treatment [3,16]. Eight studies were observational [4,7,12,13,14,15,17,19], one study was a case-control study [3], and two studies were a non-randomized clinical trials [16,18].

Three out of eleven studies were carried out in Italy [4,12,13], two studies were carried out in Korea [7,14], and two studies were carried out in France [16,18]. The remaining four studies were performed in Turkey [19], Brazil [15], Spain [17], and jointly in Italy and Australia [3]. Two studies [12,19] were carried out in a University hospital, three studies were carried out in a tertiary referral hospital [4,14,16], one study was carried out in the emergency department [6], and five studies did not report information about the setting [3,13,15,17,18]. MacDougall et al. [3] reported that both HIMP and SHIMP gains discriminated patients with UV and BV from healthy controls with 100% sensitivity and 100% specificity [2]. Shen et al. [18] reported that SHIMPs discriminated acute UV patients from healthy controls with 100% sensitivity and 100% specificity and chronic UV patients from healthy controls with 87% sensitivity and 83% specificity. Furthermore, they reported that both the HIMP and SHIMP [18] discriminated BV patients from healthy controls with 100% sensitivity and 100% specificity. Park et al. [7] reported that the mean SHIMP VOR gain discriminated the affected from the healthy sides in acute VN patients with 95% sensitivity and 91% specificity. Eight out of eleven studies reported the ages of the enrolled patients [4,12,13,14,18,19], and five out eleven reported the sex [4,12,13,14,18].

## 4. Discussion

This review aimed to explore the clinical application of the SHIMP in the diagnosis of vestibulopathy in both the acute and chronic phases. All the included studies reported an alteration of the VOR gain in both UV and BV. The SHIMP was compared with other clinical and instrumental tests to diagnose BV [3,13,15,16,17,18] and UV [3,4,7,12,14,15,17,18,19]. Four studies [4,14,16,19] reported that the VOR gain can be suitably assessed with the SHIMP in a hospital setting and one [7] reported similar results in an emergency department. Furthermore, four studies included patients evaluated in the acute phase [4,7,12,18], suggesting that VOR gain evaluation with the SHIMP could be a valuable tool for the clinician in the acute phase in differentiating vestibulopathy from other forms of dizziness and vertigo.

Rey-Martinez et al. [17] provided significant evidence for the influence of predictability on the delay of SHIMP saccadic responses. Even though predictability had an effect on saccadic latency for the SHIMP method, this effect could have an impact on the test’s main outcome. Furthermore, a lower SHIMP VOR gain in combination with a lower SHIMP overt saccade prevalence is more likely to predict the onset of chronic symptoms and the need for a rehabilitation assessment [12]. The SHIMP could also be a useful tool for the vestibular assessment of the VOR slow phase and to evaluate the visually enhanced vestibulo-ocular reflex (VVOR) and vestibulo-ocular reflex suppression (VORS) [15]. Indeed, by proving the connection between the lesioned peripheral vestibular system and the central nervous system’s adaptation process, VVOR and VORS testing might be added to the standard video head impulse test procedure. The vestibular loss can be efficiently cancelled, and retinal slip during head movements can be reduced by substituting another type of eye movement system [20]. Corrective saccades are then used as part of an adaptive technique to supplement the VOR’s slow-phase component for vestibular rehabilitation [21]. When the goal is a visual fixation on a stationary earth-fixed dot (HIMP), any passive impulsive head rotation generated in healthy people is eye rotation in the skull suitable for stabilizing the retinal image [2]. In contrast, if the intended goal is a fixation on a target moving with the head (SHIMP), eye rotation in the skull is not completely sufficient for stabilizing the retinal image. Indeed, at the end of the head rotation, the eyes of the people being examined ae on the dot and, at that point, a rapid, saccadic, ocular movement must be used to bring the eyes back to the target, which is head-fixed. We termed this phenomenon as the visuo-vestibular interaction [4] and noticed that, in each paradigm, HIMP vs. SHIMP, the eye movements are quite similar; both response patterns remain suitable for the common goal of stabilizing the chosen retinal image [2].

Menière’s disease was considered in two studies that enrolled 20 patients with UV [18,19]. Patients with Menière’s disease treated with intratympanic gentamicin injections were considered chronic unilateral vestibulopathy patients with a reduction of HIMP VOR gain values. These results are consistent with the study by MacDougall et al. [3] in which altered SHIMP and HIMP gain values were obtained in patients with bilateral and unilateral vestibular loss. The authors [3] affirmed that the SHIMP encourages a more exact estimation of the VOR pick-up by killing the larger part of the catch-up saccades during the test. Shen et al. [18] affirmed that the SHIMP can also be utilized to decrease estimation mistakes of the VOR pick-up resulting from covert saccades and spontaneous nystagmus. After the SHIMP was introduced, the peak saccade velocity and frequency of saccadic response have been suggested as alternative parameters. Indeed, peak saccade velocity could be a useful parameter in identifying patients with acute VN by generating SHIMP saccades during head rotation. Other tests, such as HIMP VOR gains and the caloric test, may not be able to explain what SHIMP VOR gains and peak SHIMP saccade velocity could reveal [7]. Lee et al. suggested the importance of the SHIMP parameters in the symptom recovery of VN. Indeed, the anti-compensatory saccades of SHIMPs do not appear during vestibular compensation in the recovery phase of an acute VN, and they might predict that residual symptoms could remain in the chronic phase. On the other hand, patients demonstrating rapid VOR gain recovery at 1 month are more likely to be symptom-free at 6 months [14].

To the best of our knowledge, no studies have systematically reported the clinical usefulness of the SHIMP during clinical practice. However, from a thorough examination of the literature about the usefulness of the new paradigm being used in daily clinical practice, a good amount of information comes to the aid of the clinician. It is an acquired notion that the HIMP is a useful paradigm in the evaluation of the otoneurological patient [22,23], both in the acute and chronic stages. We hypothesized that recent literature could also support this theory for the SHIMPs paradigm with high diagnostic accuracy [3,7,18]. Furthermore, another important point to consider is the possible diagnostic use of the SHIMP in the early stage of the vestibular symptoms. Indeed, three studies used the two paradigms in the first 72 h from the onset of symptoms [4,7,14]. Recently it has been demonstrated [4] how the SHIMP paradigm provides useful information about the value of the visual (saccadic system)–vestibular interaction as a new recovery strategy in patients with VN. This is an important clinical point because no analogous indicators exist in other vestibular assessments in common clinical use, such as caloric testing or vestibular evoked myogenic potentials [24,25]. Indeed, the reappearance of the SHIMP saccades is triggered by even the slightest recovery of the VOR that takes the patient’s eyes out of the target, initiating the restoration of the synergy of slow and saccadic eye movements. Compared to the HIMP, in patients with vestibulopathy, the SHIMP shows a reversed saccadic pattern. HIMPs elicit compensatory saccades opposite to the direction of head rotation. In contrast, SHIMPs elicit anti-compensatory saccades in the direction of head rotation in healthy people.

## 5. Limitation

The clinical and methodological heterogeneity of the included studies made it impossible to conduct a quantitative summary of results; second, the included studies showed a high degree of heterogeneity in terms of the time since the onset of symptoms, the setting in which the assessment was performed, the ages and genders of the patients, drug assumptions, and the comparison with other instrumental evaluations.

## 6. Conclusions

The SHIMP could be a useful tool to diagnose a VOR alteration in patients with unilateral and bilateral vestibulopathy. Further well-designed studies are needed to evaluate if the new paradigm could replace the HIMP in both the acute and chronic phases of vestibulopathy.

## Figures and Tables

**Table 1:** Demographic and Clinical Characteristics of Participants.

Number of Participants, Etiology, and Diagnostic Accuracy						
Author, Year	Unilateral Vestibulopathy	Bilateral Vestibulopathy	Comparators	Age	Sex	NOS Score
Casani AP., 2021 [12]	30 Unspecified.	NA	HIMP	Mean 61.50 ± 17.74	19 F, 11 M	7
Malara P., 2021 [13]	NA	1 Neuroborreliosis.	HIMP, VEMPs	72	1 F	3
Manzari L., 2020 [4]	15 Vestibular Neuritis.	NA	HIMP	Mean 58.73 ± 10.73	6 F, 9 M	6
Park JS., 2020 [7]	21 Vestibular Neuritis, 95% sensitivity, 91% specificity.	NA	HIMP	NA	NA	6
Lee JY., 2020 [14]	27 Vestibular Neuritis.	NA	HIMP	Mean 56.37 ± 12.69	17 M, 10 F	6
Kirazli G., 2020 [19]	3 Vestibular Neuritis, 7 Menière’s disease.	6 Idiopathic.	HIMP, fHIT	Mean 57.33 ± 6.53 (Bilateral); 50.30 ± 10.02 (Unilateral)	NA	6
Ramos BF., 2019 [21]	20 Vestibular Neuritis.	3 Idiopathic	HIMP, VVOR, VORS	Ranged from 20 to 75 years.	NA	4
de Waele C., 2017 [16]	NA	3 Gentamicin, 1 Genetic, 4 Idiopathic.	HIMP	Mean 56 ± 16;	NA	4
Rey-Martinez J., 2017 [17]	95 Unspecified.	NA	HIMP	Mean 48.44 ± 2.65	NA	7
Shen Q., 2016 [18]	13 Menière’s disease, 38 Operated schwannoma. 100% sensitivity and 100% specificity in the acute phase. 87% sensitivity and 83% specificity in chronic phase.	6 Idiopathic 100% sensitivity and 100% specificity.	HIMP, Caloric	Mean 58 ± 13	34 M, 23 F	7
MacDougall HG., 2016 [3]	5 Operated schwannoma. 100% sensitivity and 100% specificity.	2 Gentamicin, 3 Idiopathic. 100% sensitivity and 100% specificity.	HIMP	NA	NA	5

NA = Not Applicable; NOS = Newcastle–Ottawa Scale; HIMP = head impulse paradigm; VEMPs = vestibular evoked myogenic potentials; fHIT = functional head impulse test; VVOR = visually enhanced vestibulo-ocular reflex; VORS = vestibulo-ocular reflex suppression; DA = diagnostic accuracy.

## Data Availability

Not applicable.

## Appendix A

Adapted version of the Newcastle–Ottawa scale used in the present study for quality assessment (maximum 7 stars).
